# Transcranial magnetic stimulation input–output curve slope differences suggest variation in recruitment across muscle representations in primary motor cortex

**DOI:** 10.3389/fnhum.2024.1310320

**Published:** 2024-02-07

**Authors:** Lari M. Koponen, Miles Martinez, Eleanor Wood, David L. K. Murphy, Stefan M. Goetz, Lawrence G. Appelbaum, Angel V. Peterchev

**Affiliations:** ^1^Department of Psychiatry and Behavioral Sciences, Duke University, Durham, NC, United States; ^2^Centre for Human Brain Health, School of Psychology, University of Birmingham, Birmingham, UK; ^3^Center for Cognitive Neuroscience, Duke University, Durham, NC, United States; ^4^Department of Electrical and Computer Engineering, Duke University, Durham, NC, United States; ^5^Department of Neurosurgery, Duke University, Durham, NC, United States; ^6^Department of Psychiatry, University of California, San Diego, San Diego, CA, United States; ^7^Department of Biomedical Engineering, Duke University, Durham, NC, United States

**Keywords:** transcranial magnetic stimulation, TMS, motor evoked potential, MEP, input–output curve, IO curve

## Abstract

Measurement of the input–output (IO) curves of motor evoked potentials (MEPs) elicited by transcranial magnetic stimulation (TMS) can be used to assess corticospinal excitability and motor recruitment. While IO curves have been used to study disease and pharmacology, few studies have compared the IO curves across the body. This study sought to characterize IO curve parameters across the dominant and non-dominant sides of upper and lower limbs in healthy participants. Laterality preferences were assessed in eight healthy participants and IO curves were measured bilaterally for the first dorsal interosseous (FDI), biceps brachii (BB), and tibialis anterior (TA) muscles. Results show that FDI has lower motor threshold than BB which is, in turn, lower than TA. In addition, both BB and TA have markedly shallower logarithmic IO curve slopes from small to large MEP responses than FDI. After normalizing these slopes by their midpoints to account for differences in motor thresholds, which could result from geometric factors such as the target depth, large differences in logarithmic slopes remain present between all three muscles. The differences in slopes between the muscles could not be explained by differences in normalized IO curve spreads, which relate to the extent of the cortical representation and were comparable across the muscles. The IO curve differences therefore suggest muscle-dependent variations in TMS-evoked recruitment across the primary motor cortex, which should be considered when utilizing TMS-evoked MEPs to study disease states and treatment effects.

## 1 Introduction

Transcranial magnetic stimulation (TMS) of the human motor cortex is commonly used to investigate corticospinal tract integrity. Although TMS has been approved for medical treatments and has been extensively used in research to study neurological diseases and the influence of medications on the body, few studies have systematically tested the impact of TMS on different muscles throughout the healthy human body.

A key region of study for understanding cortical responses to TMS is the primary motor cortex (M1). Within M1, the area corresponding to the first dorsal interosseous (FDI) muscle on the dominant side has been widely tested due to its superficial location on the cortex, relatively low motor threshold, and ease-of-measurement with a simple surface electromyography (EMG) montage on the hand. The simplest method of probing the FDI and corresponding cortex is through identification of the “motor hotspot,” an area when stimulated that activates the FDI (Wassermann et al., 1992; Pascual-Leone et al., 1994). FDI stimulation, in turn, is used for subsequent dosing of most clinical protocols, leading to standardization of procedures that allow for consistency and replicability of responses (Rossi et al., 2021).

Despite the widespread use of MEPs, they are a relatively coarse measure due to the broad distribution of the stimulating TMS electric field, variability in the underlying neuroanatomy, and individual differences in motor system responsiveness. Further, MEP responses to identical TMS pulses have large, random variability (Goetz et al., 2014). As such, characterizing the average MEP response to a range of TMS intensities can provide insight into the functionality of muscles. In particular, the relationship between stimulation intensity and average MEP amplitude, or the input–output (IO) curve, can be used as a measure of corticospinal excitability (Ray et al., 2002) that may differ between muscles due to their location on the body (Penfield and Boldrey, 1937; Roux et al., 2020) and their typical usage (Nudo et al., 1996; Cardinali et al., 2012). Notably, within the same muscle, the IO curve slope is remarkably consistent across healthy volunteer participants of different ages (Pitcher et al., 2003), but can change due to, for example, a spinal cord injury (Nardone et al., 2015) or the phase of the ongoing cortical μ-rhythm (Schaworonkow et al., 2019). The slope can also be either reduced or increased by repetitive TMS depending on the repetition rate (Gangitano et al., 2002; Houdayer et al., 2008). Such variations in cortical muscle representation and excitability prompt the question of how responses to TMS differ across the body, and particularly in relationship to laterality dominance, so-called handedness and footedness.

Laterality dominance greatly affects usage, even in the same muscle (Wassermann et al., 1992; Triggs et al., 1994) and is associated with asymmetric neural control. As a result, muscle representations, and responses to TMS may differ and there is currently no strong consensus on how TMS differentially affects the two motor cortices. When measuring IO curves, some investigations have found steeper slopes on the non-dominant side (Daligadu et al., 2013; Bracco et al., 2017), while others have found no difference between dominant and non-dominant hemispheres (Davidson and Tremblay, 2013; Smith et al., 2017; Dharmadasa et al., 2019). The steepness of the IO curve reflects the range of TMS strengths necessary to raise muscle activity from inactivity to saturation, so an increased slope may be reflective of increased excitability or decreased inhibition (Daligadu et al., 2013).

Many past studies exploring lateralization of excitation have focused on the upper body, particularly on the FDI, with less research addressing excitability of the lower body (Smith et al., 2017; Dharmadasa et al., 2019). TMS studies of lower extremities tend not to also test upper extremities, due to higher motor threshold needed to elicit responses in the lower body, and consequently the need for different TMS coils. The primary reason for the higher motor thresholds lies in the neuroanatomy: the muscle representation areas are “deeper”; that is, the distance from the scalp to the relevant cortical targets are larger for the lower extremities (Kesar et al., 2018) resulting in a weaker induced electric field at a given TMS intensity (Stokes et al., 2013). As a result, beyond early TMS research with non-focal circular coils (Brouwer and Ashby, 1990; Chen et al., 1998), past studies have not attempted to directly compare upper and lower limb IO curves which likely differ considerably due to differences in fine motor control for these muscles.

Therefore, the purpose of this study was to investigate differences in excitability, bilaterally, and across upper and lower body muscles, by obtaining IO curves within the same experimental session. For this purpose, three muscles were targeted bilaterally, two upper extremity and one lower extremity, and information was collected from all participants about their hand and foot dominance preferences. It was hypothesized that excitability, measured through IO profiles would be greater for the upper-than-lower muscles and would be greater for the dominant side of the body.

## 2 Materials and methods

### 2.1 Participants

Eight healthy young adults (5 females; age 21−28 years, mean 24 years) completed the experimental protocol. The number of participants was justified by the large effect size (Cohen’s *d* > 1.0) expected based on our pilot experiment with one participant, where all muscles other than FDI had IO curve slopes well outside of the values for FDI in this subject and in prior data (Peterchev et al., 2013), and the study design with 3 muscle pairs measured for each participant: An experiment with 8 participants (i.e., 16 observations of any given muscle from either side of the body, or 24 muscle pairs for lateralization) would be sensitive to Cohen’s *d* = 1 with >99% power at α = 0.05 and can reliably detect effect size of Cohen’s *d* = 0.60 with 80% power. Participants were excluded if they had any contraindications to TMS, as indicated by verbal screening of the transcranial magnetic stimulation adult safety screen (TASS) (Keel et al., 2001). Informed consent was obtained from all participants, and they were compensated $20/hour for their time. Study procedures were approved by the Institutional Review Board of Duke University Health System and conformed to the Declaration of Helsinki.

### 2.2 Design

Participants completed a single study session lasting between 2 and 3 h. During the study, participants were first informed about the experimental protocol and provided informed consent to participate. Then, the participants filled out the revised Waterloo handedness and footedness questionnaires (Elias et al., 1998). Next, electromyography (EMG) electrodes were applied, and the stereotaxic neuronavigation system was co-registered to participants heads to allow for real-time coil placement guidance and offline measurement of the spatial precision of TMS delivery. Following this, TMS motor hotspots were identified using visual detection of muscle twitches and online EMG measurement for the first dorsal interosseous (FDI), biceps brachii (BB), and tibialis anterior (TA) muscles, bilaterally. To help find the TA hotspot, EMG was also recorded from the vastus lateralis on the left and right quadriceps femoris (QF) muscle. IO curves for QFs were not acquired as, based on our pilot experiment, these could not be derived without changing the coil or pulse properties due to limited maximum stimulator output. Once these six motor hotspots were found, and their intensity and location parameters recorded, IO curve sampling was performed for each target muscle according to a randomization procedure, counterbalanced across participants. Participant comfort was assessed verbally throughout the experiment, and breaks were offered and given whenever participants desired. These methods and subsequent analyses are each described in greater detail in the following sections.

### 2.3 Electromyography (EMG)

**FIGURE 1:**
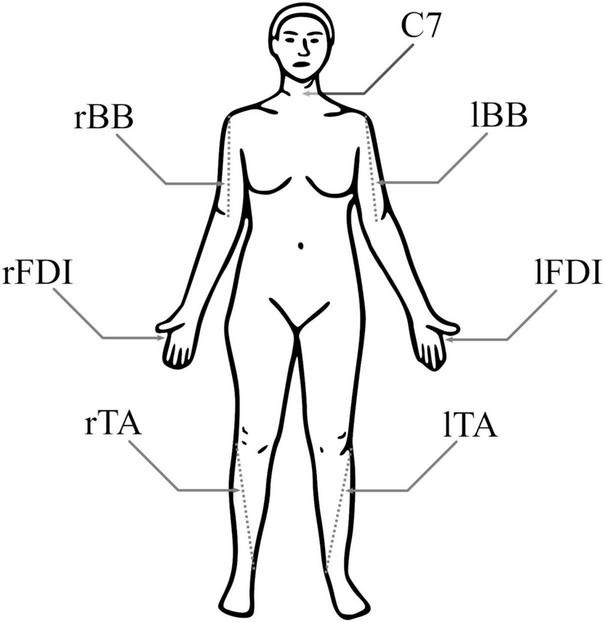
Electromyography (EMG) electrode positions. Both biceps brachii (BB) had a pair of electrodes around the 1/3 point on a line from fossa cubit to medial acromion, both first dorsal interosseous (FDI) had pair of electrodes over the belly of the muscle and the proximal phalanx of index finger, and both tibialis anterior (TA) had a pair of electrodes around the 1/3 point on a line between the tip of fibula and the tip of medial malleolus. For each muscle, the negative electrode was more medial. The reference electrode was at C7.

Participants were seated comfortably in a chair, given a pillow to place on their lap, and instructed to relax their limbs. Skin preparation at each electrode location was performed using exfoliant gel and alcohol wipe pads; 24 mm silver/silver-chloride surface electrodes (Kendall H124SG, Covidien, Ireland) were placed bilaterally in a bipolar belly–tendon montage over the FDI muscles and in a bipolar belly-belly montage over the TA and BB muscles (Figure 1). Electrodes were placed following SENIAM recommendations (Hermens et al., 2000) and scaled for each participant based on the length of their limbs. The reference electrode was placed over the C7 vertebra. EMG signals were band-pass filtered between 0.1 and 1,000 Hz and recorded with a multi-channel bipolar amplifier with an integrated analog-to-digital converter (BrainAmp ExG, BrainVision, USA) with a 5,000 Hz sampling rate.

Because volitional and spontaneous muscle activity can influence corticospinal excitability, participants were asked to relax their muscles during data collection. EMG activity was monitored during the experiment with a custom MATLAB toolbox (Supplementary Figures 1, 2), and if participants displayed spontaneous muscle activation during the experiment, they were instructed to re-relax their muscles. Further, if more spontaneous activity began to build up between pulses, or it was seen in the real-time data monitor in a 100 ms time-window prior to a TMS pulse, data collection was paused and participants were asked to voluntarily activate their muscles to relieve pre-activation (i.e., shake the limb briefly). Any trial where pre-activation exceeded 50 μV peak-to-peak in the 100 ms time-window prior to a pulse was subsequently omitted from analysis. A total of 97 out of 3,637 trials (2.7%) were rejected due to pre-activation.

### 2.4 Stereotaxic neuronavigation

Coil position and orientation relative to the head was monitored with a stereotactic neuronavigation system (Brainsight, Rogue Research, Canada), which measured coil location with six degrees of freedom (three spatial and three angular coordinates). At the time of each TMS pulse, a trigger was sent to the neuronavigation system to record the location of the coil. As the primary purpose of neuronavigation was to ensure repeatable coil location between the mapping and the IO curve acquisition, individual MRI images were not acquired and the neuronavigation was performed using built-in MNI template head, scaled to approximate individual head sizes. For M1, for each participant, the surface of the scaled template head was within 2−5 mm of participant’s scalp.

### 2.5 TMS motor hotspot identification

MEPs were evoked from all muscles by monophasic pulses delivered with a bent D-B80 figure-of-eight coil connected to a MagPro X100 with MagOption stimulator (MagVenture, Denmark). The stimulator was set to “power mode” which increased the duration of the initial rising coil current by about 40%. The coil current direction was set to “reverse” which induces a posterior–anterior oriented current in the brain for monophasic pulses and posterior-pointing coil handle. Motor hotspots were defined as the optimal coil location to elicit maximal MEP responses with the lowest stimulator intensity. Consistency and accuracy over each hotspot were maintained using stereotaxic neuronavigation.

For each participant, hotspots were localized with the following procedures. First, the stimulator output was set to 40% maximum stimulator output (MSO), which was expected to be above the motor threshold for FDI. Then, the search for the FDI hotspot was started roughly 75% of the way from the ear to midline following the central sulcus in the MNI template. If no FDI MEP was elicited, stimulator intensity was increased by 10% MSO. Once an FDI MEP was elicited, confirmed by both EMG and visual observation of the correct finger motion, intensity was lowered until a 100−1000 μV peak-to-peak amplitude MEP was elicited. This preliminary hotspot location was then marked on the neuronavigation system, and nearby locations were systematically probed to localize the true hotspot. For this refinement, pulses were probed 5 mm in the dorsal, ventral, anterior, and posterior directions, each followed by probe at the preliminary hotspot location, each with a 4−6 s interstimulus interval. If a probed location elicited a stronger response, it was set to be the new preliminary hotspot and the process was repeated. Between repetitions, the intensity was adjusted to keep the responses between 100−1000 μV. Once this process converged, the coil orientation was optimized by testing an 80° range of different coil orientations in 20° rotational increments and the one with largest response was selected. If the optimum orientation was at the edge of the tested range, a new range of coil orientations around the new optimum orientation was tested. This process was repeated until convergence. The final orientation with the largest response was saved, and the final mapping intensity was denoted as the approximate midpoint intensity (MI). Once the FDI hotspot was established, a similar procedure was used for BB and then TA on the same hemisphere, and then for all three MEPs on the opposite hemisphere starting again from the FDI. The left hemisphere was mapped first for all subjects, followed by the right hemisphere.

### 2.6 Input–output curve acquisition

Once all hotspots were determined by the above procedure, IO curves were sampled at each location. To mitigate any order effects, the order of sampling was counterbalanced between participants, and it proceeded as upper body, lower body, then upper body, interleaved between the dominant and non-dominant sides. Within each muscle, the MEP response to a given TMS pulse has both stimulation-intensity dependent random variability (Goetz et al., 2014) and hysteresis based on the previous MEP response (Möller et al., 2009). To accurately estimate an IO curve, both effects must be considered. To ensure practical sampling of multiple IO curves, we implemented an efficient IO curve sampling routine in our custom MATLAB toolbox (Supplementary Figure 2): Before sampling each IO curve, the maximum tolerable stimulation intensity was determined, with a preset upper limit of MI+25% MSO. Then, we generated a randomized sequence of pulses from MI−25% MSO to the maximum tolerable intensity (two samples at each intensity from MI−20% MSO to MI+10% MSO, one sample at all other intensities), where the randomized sequence converts hysteresis into additional random variability. This sampling strategy is a combination of variable sampling density (Peterchev et al., 2013), where most pulses are used to measure the transition region of the IO curve, and single-pulse-per-intensity sampling (Nieminen et al., 2019), which maximizes the resolution for the midpoint determination (to reach the desired total number of pulses, we sampled two pulses at each intensity near MI as our TMS device did not support 0.5% intensity increments). With this sampling, 22 TMS pulses are delivered in each ± 5% MSO intensity window near MI. Depending on the maximum tolerable intensity, this sampling process resulted in 72−82 samples per IO curve which took roughly 7 min per muscle with a randomized 4−6 s interstimulus interval. The range of intensities and the number of pulses per intensity were tested to ensure a reliable estimate of IO curve parameters for a population of 100 virtual participants generated with the statistical model of MEP amplitudes (Goetz et al., 2019), and the chosen range of intensities is similar to a prior study (Peterchev et al., 2013). Notably, whilst this sampling scheme worked very well for the FDI, it had some limitations for the other two muscles: Optimal sampling scheme for a shallower IO curve needs more high-intensity pulses to accurately estimate the saturated MEP response. In each randomized sequence, the instantaneous maximum allowed value for stimulator output tapered in over the first quarter of pulses to avoid very strong stimuli at the beginning of the IO curve sampling. Neuronavigation was monitored to ensure accurate coil positioning throughout IO curve sampling. For the analysis, any trial where the coil position deviated from the hotspot by more than 5 mm was excluded (which removed 4 pulses across the whole experiment) or where the coil orientation deviated by more than 10° (which removed the last 20 pulses from the IO curve of dominant TA for participant 4, when the coil had accidentally been aimed 20° clockwise from the intended direction after a pause during IO curve acquisition). After removal of these 24 pulses, the root-mean-square distance to the target was 0.9 mm and the root-mean-square orientation error was 1.9°.

### 2.7 Input–output curve model

For each muscle, a least-squares sigmoidal IO curve fit was computed as


(1) $$\log_{10}\frac{V_{\text{MEP}}\,\left(x\right)}{V_{\text{MEP}}\,\left(0\right)}=\Delta \,y\cdot \frac{1+\text{erf}\,\left(\sqrt{\pi }/20/\Delta \,y\cdot s\cdot \left(x-m\right)\right)}{2},$$

where *V*_MEP_(*x*) is the MEP amplitude at simulator output, *x*, and erf is the Gauss error function (Peterchev et al., 2013). The three degrees of freedom were the ratio between noise floor and a saturated MEP Δ*y*, slope *s* in dB/%MSO, and midpoint *m*. The log-transformation of the MEP amplitudes removes the differences arising due to different EMG montages and helps improve the normality of the amplitude distribution (Nielsen, 1996). The multiplicative factor $\sqrt{\pi }/20/\Delta \,y$ inside the erf ensures desired units for the fitted slope.

First, the baseline peak-to-peak MEP amplitude in the absence of TMS pulses, *V*_MEP_(0), was estimated from the pre-stimulus EMG data. Then, an iterative-least-squares fit was computed starting from Δ*y* = max⁡log_10_⁡*V*_MEP_/*V*_MEP_(0), *s* = 1 dB / %MSO (a very shallow IO curve), and *m* = MI (the approximate midpoint intensity from the mapping). Of the resulting 48 IO curves (Supplementary Figure 3), two were rejected after visual inspection: for one curve the range of stimuli did not encompass the fitted midpoint, the other failed to conform to data resulting in an “infinite” slope. The remaining IO curves were evaluated by comparing their midpoints, slopes, and normalized slopes, i.e., the product of the slope and the midpoint. This normalization removes the arbitrary constant factor arising from the MSO of the TMS equipment and has been previously used to remove the effect of different pulse waveforms on the FDI IO curve slope (Peterchev et al., 2013). For two point-like muscle representations, this normalization would exactly cancel out the effect of the different induced electric field at the two targets for the same stimulator output setting. Therefore, as the muscle representations are relatively small (Nieminen et al., 2019; Weise et al., 2019) compared to the spread of the TMS-induced electric field (Deng et al., 2013), this normalization will approximately cancel the difference in IO slope arising from the different depth of the cortical targets. As such, this makes the normalized slope approximately invariant to both the TMS equipment and the variation caused by differences in the motor threshold due to differences in the depth of the cortical muscle representation.

**FIGURE 2:**
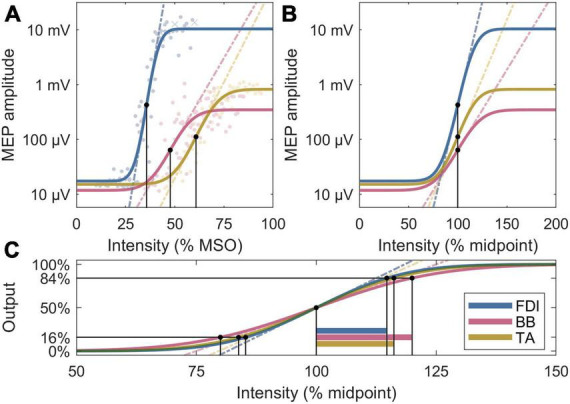
Four analysis metrics visualized for three IO curves whose parameters are closest to the participant averages. **(A)** Midpoints are denoted by black dots, and slopes by dashed lines. The individual MEPs are denoted by colored dots or crosses, for accepted and rejected samples, respectively. **(B)** Normalized x axis: Normalized slopes are denoted by dashed lines. **(C)** Normalized x and y axes: Normalized recruitment spreads are denoted by horizontal bars; their multiplicative inverses are proportional to the slopes of the dashed lines, which exclude the effects of the lower and upper plateau levels seen in panels **(A,B)**.

To explain the observed differences in the normalized slopes, *post-hoc*, we further removed the ratio between noise floor and a saturated MEP, Δ*y*, to obtain the normalized recruitment spread. Assuming identical activation threshold for individual neurons across the M1, this metric is sensitive to the extent of a cortical representation, with a larger spread corresponding to a broader representation area, since the cortical volume exposed to a suprathreshold electric field increases with the pulse intensity. To facilitate direct comparison to the slopes, the multiplicative inverse of the spread is shown as, $\sqrt{2}\cdot m\cdot s\cdot \sqrt{\pi }/20/\Delta \,y$, which corresponds to the ratio of the midpoint and the width of the transition region of the error function in Equation (1). The IO metrics are visualized in Figure 2.

### 2.8 Statistical analysis

The IO curve parameters were compared with a linear mixed-effects model (LME). We fitted a model with a fixed-effect intercept, muscle (FDI, BB, or TA) and its lateralization (dominant, non-dominant) as fixed effects, and the participant as a random effect. The fit was computed with “fitlme” and the statistical inference with “coefTest” (MATLAB R2021a, MathWorks, USA). The three possible contrasts between the muscles were corrected for multiple comparisons with the Bonferroni–Holm method (Holm, 1979).

To validate that our main finding on the normalized slope is robust, as a *post-hoc* analysis we pooled our dominant FDI data with an earlier dataset and compared this data to each individual muscle with a separate two-tailed independent two-sample *t*-test. To facilitate interpretation of these tests, we further report the false positive risk (FPR) for each *t*-test (Colquhoun, 2014, 2019).

## 3 Results

Of the eight participants, the Waterloo handedness score suggests that seven were right-handed and one left-handed. The Waterloo footedness score suggests that five participants were right-footed, one left-footed, and two had no preference for footedness. The TA data for these two was omitted from the comparisons. For each participant, we localized the motor hotspot for all six muscles using stereotaxic neuronavigation with the MNI template head model. Despite using a template head, each hotspot was found near the template central sulcus in the posteroanterior direction. All hotspots were in the expected lateromedial order, with FDI most lateral and TA most medial. The optimum direction for all FDI and BB muscles was always approximately normal to the central sulcus of the template head, between anterior and anteromedial directions. The optimum direction for the TA was both more lateral and broader, with the optimum between anterior and anterolateral directions and large responses extending all the way to the lateral direction.

**FIGURE 3:**
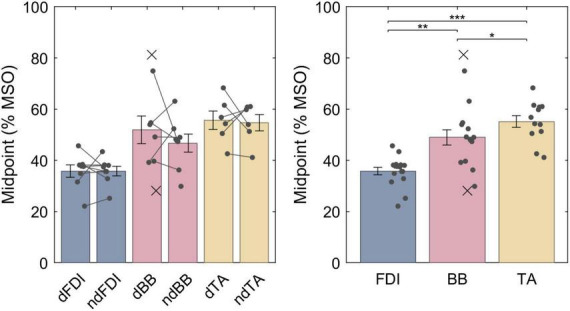
The IO curve midpoints for different muscles. Symbol “d” denotes the dominant side and “nd” the non-dominant side. The data from individuals is shown with either a dot or a cross, for included and rejected data, respectively. In the left panel, a gray line connects each included pair of midpoints. The error bars denote standard error which assumes normal distribution, and “*,” “**,” and “***” denote statistically significant differences at *p* < 0.05, *p* < 0.001, and *p* < 0.000001, respectively. FDI had lower midpoint than BB which had lower midpoint than TA.

The LME model fit for the midpoints was, respectively, FDI: 35.8% MSO (95% confidence interval 30.9−40.7% MSO), BB: 48.4% MSO (43.4−53.5% MSO), and TA: 55.3% MSO (49.9−60.7% MSO), and the difference between the midpoint on the dominant and non-dominant side was 1.5% MSO (−2.5 − +5.5% MSO). For a full list of LME model coefficients, see Supplementary Table 1. As expected from the literature, the resting motor threshold, and consequently the IO curve midpoint, was lower for FDI than for either BB (Bonferroni–Holm corrected *p* = 9.1 ⋅ 10^−6^) or TA (*p* = 7.4 ⋅ 10^−9^) (Figure 3). Further, the BB had a lower threshold than TA (*p* = 0.013). There was, however, no observable effect with lateralization (*p* = 0.46).

**FIGURE 4:**
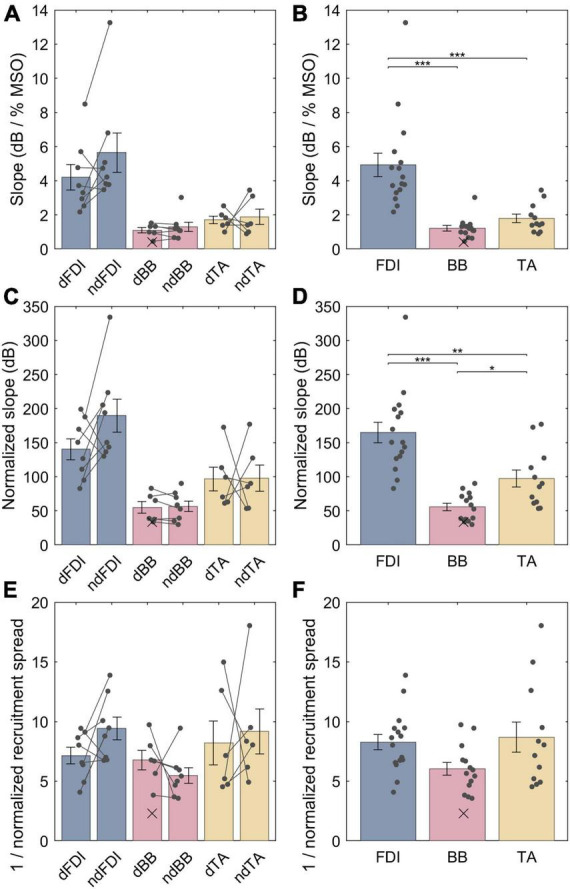
The IO curve slopes and recruitment spreads for different muscles. **(A,B)** Non-normalized slope. **(C,D)** Normalized slopes. **(E,F)** Multiplicative inverse of normalized recruitment spread. The data from individuals is shown with either dots or crosses, for included and rejected data, respectively. Two datapoints were rejected; the other rejected datapoint with an “infinite” slope lies outside the plot range. In panels **(A,C,E)**, gray lines connect each included pair of muscles. The error bars denote standard error which assumes normal distribution, and “*,” “**,” and “***” denote statistically significant differences at *p* < 0.05, *p* < 0.001, and *p* < 0.000001, respectively. The FDI IO curves had notably steeper slopes than either BB or TA, and the TA had steeper normalized slopes than BB. Unlike slopes, normalized recruitment spreads did not differ significantly between the muscles.

The LME model fit for the non-normalized slopes for FDI, BB and TA were, respectively, 4.9 (3.9−5.9), 1.3 (0.28−2.3) and 1.7 (0.57−2.8) dB / % MSO; the difference between the dominant and the non-dominant side was −0.57 (–1.5 − +0.34) dB / % MSO. For a full list of LME model coefficients, see Supplementary Table 2. That is, the FDI IO curves had much steeper non-normalized slopes than either BB (*p* = 1.2 ⋅ 10^−7^) or TA (*p* = 2.1 ⋅ 10^−6^) (Figures 4A, B), whereas no difference was observed between BB and TA (*p* = 0.54) or with lateralization (*p* = 0.21). The non-normalized slopes, however, are biased by different “depth” of cortical representation. Thus, for an unbiased comparison, the IO curve slopes needed to be normalized by multiplying them with their respective midpoints. As described in section “2.7 Input–output curve model,” this removes the arbitrary constant factor arising from different TMS-induced electric field at different depths at a given stimulator output setting. The LME model fit for the normalized slopes for FDI, BB and TA were, respectively, 165 (143−187), 54 (31−78), and 97 (72−122) dB; the difference between the dominant and the non-dominant size was −20 (−47 − +7.0) dB. For a full list of LME model coefficients, see Supplementary Table 3. Overall, normalizing for the differences in the IO curve midpoints reduced, but did not remove, the difference between FDI and the other two muscles. The FDI IO curve had much higher slope than either BB (*p* = 6.3 ⋅ 10^−8^) or TA (*p* = 3.7 ⋅ 10^−4^) despite the normalization by the midpoint (Figures 4C, D). Further, BB had a statistically significantly lower slope than TA (*p* = 0.015). For each muscle, the dominant side had shallower IO curve. This trend was, however, not statistically significant (*p* = 0.14). Notably, as expected when removing a random bias source, the normalization of slopes improved the model fit and greatly reduced the amount of unexplained variance in the model (Supplementary Tables 2, 3). Finally, the normalized recruitment slopes (Figures 4E, F) showed much less variability between different muscles, and despite a good model fit with similar amount of unexplained variance as the model for the normalized slopes there were no statistically significant differences between any of the three pairs of muscles (FDI–BB, *p* = 0.076; FDI–TA, *p* = 0.71; BB–TA, *p* = 0.071) nor lateralization (*p* = 0.43). For a full list of LME model coefficients for the normalized recruitment slopes, see Supplementary Table 4.

The LME models provide estimates of the population mean of each parameter. The individual observations of the parameters, for example, the normalized slopes, have a broad range around this mean (Figures 4C, D): On the dominant size of the body, the full range of observations for the normalized slope for the FDI ranged from 83 to 200 dB (mean 140 dB ± standard deviation 43 dB), for BB—from 34 to 83 dB (55 ± 21 dB), and for the TA—from 61 to 170 dB (97 ± 43 dB). Similarly, for the non-dominant side, the individual observations for the normalized slopes were 130−330 (190 ± 68), 30−90 (56 ± 21), and 53−180 (98 ± 48) dB for FDI, BB, and TA, respectively.

As an empirical validation of the normalization scheme, we compared our normalized slope for the dominant FDI (140 ± 15 dB, mean ± standard error, for our 8 participants) with the normalized slope obtained in an earlier study with a different TMS system and a different, more superficial TMS coil: 139 ± 7 dB for 11 participants with 3 different pulse waveforms (Peterchev et al., 2013). The two normalized slopes are in close agreement, indicating that, in addition to pulse waveforms, the normalization compensates for using a different TMS coil with a distinct stimulation depth profile for the same cortical target in a different participant population. This comparison supports our use of the normalization to compensate for IO slope differences resulting from different depths of the cortical targets.

To assess the robustness of the reported differences between participants, we cross-validated each muscle individually against the estimate for the dominant FDI pooled across this and our prior study (140 ± 41 dB, 19 participants total). Each individual muscle differs statistically significantly from this pooled estimate, and the differences with either of the two BB have enough evidence to ensure low false positive risk: non-dominant FDI (*p* = 0.026, *N* = 8, *FPR* = 0.13), dominant BB (*p* = 7.1 ⋅ 10^−5^, *N* = 6, *FPR* = 5.5 ⋅ 10^−5^), non-dominant BB (*p* = 1.3 ⋅ 10^−5^, *N* = 8, *FPR* = 1.0 ⋅ 10^−5^), dominant TA (*p* = 0.036, *N* = 6, *FPR* = 0.17), and non-dominant TA (*p* = 0.046, *N* = 6, *FPR* = 0.20). The relatively high false positive risk of the comparison between the dominant and the non-dominant FDI indicates that a larger sample size would be needed to reliably estimate the effect of lateralization. Simultaneously, the large difference between the dominant FDI and either of the two BB confirms, *post-hoc*, the main finding that different muscles have different normalized slopes.

## 4 Discussion

This study aimed to characterize the IO curves of three bilateral pairs of muscles to better understand how excitability manifests across the dominant and non-dominant sides of upper and lower limbs in healthy individuals. To compensate for the different EMG montages with different lead field sensitivities, the MEP amplitudes were log-transformed; and to compensate for different cortical “depths” between muscles (Kesar et al., 2018), the IO curve slopes were normalized by their midpoints. The results demonstrated significant and large differences between muscle pairs wherein both BB and TA showed shallower IO curves than those of FDI. The much shallower IO curve slopes for both BB (*s*_FDI_/*s*_BB_ = 3.8) and TA (*s*_FDI_/*s*_TA_ = 3.0) broaden the transition from subthreshold to suprathreshold MEP responses, which reduces the accuracy of MT estimation methods. Further, as MT estimation methods have been developed based on hand muscle responses, the much shallower slope may also increase the likelihood of MT misestimates similar to a case observed during our pilot study (Koponen and Peterchev, 2022). While differences were expected also between the lateralized muscles corresponding to the side of reported dominance, such differences did not reach statistical significance. The direction of the statistically non-significant lateralization trend—a steeper slope on the non-dominant side—was in agreement with previous, statistically significant, observations on FDI (Daligadu et al., 2013; Bracco et al., 2017). The small effect sizes for lateralization are in line with other previous studies (Davidson and Tremblay, 2013; Smith et al., 2017; Dharmadasa et al., 2019) where no statistically significant laterality difference were observed, and suggest that the lateralized differences in the excitability of motor representations are relatively minor and often not observable on the individual level.

The differences in the slopes were much larger between muscles than the lateralized differences. Both BB and TA had shallower normalized slope than FDI, and BB was further shallower than TA. The particularly shallow slope for BB over other muscles agrees qualitatively with the earlier work using a non-focal circular coil (Chen et al., 1998). There are several potential mechanisms that would result in a shallower IO curve. First, by log-transforming the MEP amplitudes, we ensured that these differences are not due simply to variations in the effective recording gain of the EMG electrode configurations across muscles or between individual participants. Specifically, after log-transformation, any multiplicative gain of the MEP becomes an additive offset that is subtracted out when computing the IO curve slope. One remaining interpretation would be that the shallow curves underlie representations that are either more spatially distributed or stem from areas of cortex that span larger depths that may be tangential to the surface of the scalp and thus less excitable through TMS. For such a representation, a larger range of adjustments in TMS intensity would be required to reach a saturated cortical response, and thus such IO curves should have higher normalized recruitment spreads. We did not observe such differences with the normalized recruitment spreads. Rather, the normalized recruitment spreads were comparable for all muscles. For FDI and BB, the lack of such differences was somewhat expected as both distal and proximal upper limb muscle representations are known to have comparable spatial extent when mapped at a fixed stimulation intensity (Devanne et al., 2006); our data further indicate that they also have comparable extent in depth. As such differences in spreads were not observed, the recorded differences in the slopes are unlikely to be due to differences in the geometry or extent of the cortical representations, but rather relate either to wiring differences of the corticospinal networks of the muscles within a small region of the cortex or to muscle-related variation in the dynamic range of TMS-evoked MEP responses between different muscles. As larger muscles had shallower responses than the FDI, an alternative hypothesis is that the steep response is a property of a small muscle potentially requiring a broader dynamic range of force application. Given that we only had one small muscle in our study, it is impossible to draw a definite conclusion on whether the difference in the response is of corticospinal or muscular origin from our data alone. To test this, one would need to include other small muscles in the comparison, ideally a second finger muscle and at least one small non-finger muscle. Notably, such comparison between abductor pollicis brevis for the thumb and abductor hallucis for the great toe shows different normalized slopes near motor threshold (Chen et al., 1998). Hence, the differences between muscles are unlikely to be of just muscular origin due to the size of each muscle, but rather indicate different wiring of corticospinal networks for different muscles, or of a fundamentally different distribution of individual motor unit sizes for each muscle. Interestingly, for the three tested muscles, the order of the slope corresponds to how distal the limb is on the body with the progression possibly corresponding to the degree of fine motor control, with more distal muscles being responsible for finer or faster movements, for example due to gait compensation (Tang et al., 1998). Remarkably, the statistically non-significant lateralization of the normalized slope and the normalized recruitment spread had comparable effect sizes. We hypothesize that the small differences in the spreads may largely explain the small differences in slopes between the dominant and non-dominant sides of the body. This hypothesis is supported by earlier studies that have shown that the motor representations of finger muscles are marginally larger on the dominant side (Pitkänen et al., 2015, 2018). However, given the small effect sizes, experimental validation of this hypothesis would require a larger study.

Several considerations and limitations should be noted. First, the main limitation of this study is its small sample size: While just 8 participants are enough to reliably observe that there are large differences between some muscles, much more than 8 participants would be needed to reliably study potential smaller effects between other muscles. While this study did not observe statistically significant lateralization across all three muscles, the FDI data had both steeper normalized slope and smaller normalized recruitment spread for the non-dominant side. Given the small sample size, no conclusions should be drawn from this trend. We can only conclude that the lateralized differences seen by TMS are much smaller than the differences between muscles, and that they are not apparent on the individual level. To draw a definite conclusion on lateral differences would require a much larger sample size, both in terms of the number of participants and studied muscles. Notably, in this study all IO curves were measured at rest. The resting IO curve is different from the one measured during voluntary muscle contraction (VMC). VMC both reduces the MT and increases the (unnormalized) slope of the muscle (Devanne et al., 1997) and its resting contralateral muscle pair (Smith et al., 2017). Given that larger lateralized differences have been observed during voluntary muscle contraction than at rest (Perez and Cohen, 2009), it has been hypothesized that hemispheric differences are most noticeable during response preparation, demonstrating hemispheric differences in corticospinal excitability during response preparation and execution (Reid and Serrien, 2014; Poole et al., 2018). Future studies may therefore wish to also measure the IO curves during voluntary muscle contraction, rather than just at rest, as done here. Second, the IO curve sampling in this study was to deliver one pulse at each intensity from −25% MSO to up to +25% MSO supplemented by a second pulse at each intensity from −20% MSO to +10% MSO with respect to the intensity for an approximate midpoint, defined as an intensity producing a consistent MEP between 100−1000 μV. This sampling method was designed to provide reliable IO curve estimates with a reasonably low number of stimuli, and it was tested with data drawn on the statistical model of MEP amplitudes (Goetz et al., 2019) based on data for FDI (Peterchev et al., 2013). Unsurprisingly, this sampling method worked well for both FDI in each participant but less well for their BB and TA muscles which had shallower IO curves. One IO curve for dominant BB was excluded due to a very shallow slope and estimated midpoint beyond the sampled range. This caused a bias in data; however, this bias was toward null hypothesis. Based on the data gathered in this study, to improve the IO curve parameter estimation, it is recommended that future studies either use adaptive IO curve sampling (Alavi et al., 2019, 2021) or expand the maximum intensity beyond MI+25% MSO (where tolerated by the participant) and supplement the IO curve sampling with at least 10 pulses at the maximum tolerated intensity. There is no need to supplement pulses to the lower end, as the lower plateau of the sigmoid can be readily estimated from the background EMG data. Third, localizing the TMS motor hotspot is more difficult for the lower extremities than for the hand muscles. For TA, the optimal coil orientation had large variability across participants, with the optimal orientation sometimes closer to mediolateral than posterior–anterior direction, a result in line with those reported by Hand et al. (2020) where mediolateral coil orientation had on average lower MT for TA than posterior–anterior orientation. Further, measuring several muscles simultaneously, in our case measuring both non-targeted QFs in addition to both TAs, helped to first localize the general area of the motor cortex, in line with the suggestion by Kesar et al. (2018).

## 5 Conclusions

The input–output behavior of the TMS-evoked MEPs for different muscles has large differences. Of the three tested muscles, input–output curves were shallower for BB than TA, both of which were much shallower than FDI. These effects were prominent even when the IO curves were normalized by their midpoints, which accounts for differences in the motor thresholds and related geometric factors such as the distance between the TMS coil and the muscle cortical representation. The differences in slopes between the muscles could not be explained by differences in normalized IO curve spreads, which relate to the extent of the cortical representation and were comparable across the muscles. Thus, the commonly used reference for TMS intensity, the motor representation area of the dominant FDI muscle, may not be as representative of neural recruitment even within the M1 as commonly assumed.

## Data availability statement

The original contributions presented in this study are included in this article/Supplementary material, further inquiries can be directed to the corresponding author.

## Ethics statement

The studies involving humans were approved by the Institutional Review Board of Duke University Health System. The studies were conducted in accordance with the local legislation and institutional requirements. The participants provided their written informed consent to participate in this study.

## Author contributions

LK: Conceptualization, Formal analysis, Investigation, Methodology, Resources, Software, Visualization, Writing – review & editing. MM: Conceptualization, Investigation, Methodology, Writing – original draft. EW: Conceptualization, Investigation, Writing – review & editing. DM: Conceptualization, Investigation, Writing – review & editing. SG: Methodology, Writing – review & editing. LA: Conceptualization, Funding acquisition, Investigation, Methodology, Project administration, Resources, Supervision, Writing – review & editing. AP: Conceptualization, Funding acquisition, Methodology, Resources, Supervision, Writing – review & editing.
