# Supplementary material for: Transcranial magnetic stimulation input–output curve slope differences suggest variation in recruitment across muscle representations in primary motor cortex
Source: Front Hum Neurosci. 2024 Feb 7;18:1310320. doi: 10.3389/fnhum.2024.1310320 (PMC10879434; doi:10.3389/fnhum.2024.1310320)
Supplement: Supplementary file 2 [file Data_Sheet_1.PDF]

Supplementary Material

Transcranial magnetic stimulation input–output slope differences suggest variation in recruitment across muscle representations in primary motor cortex

Lari M. Koponen*, Miles Martinez, Eleanor Wood, David L.K. Murphy, Stefan M. Goetz, Lawrence G. Appelbaum†, Angel V. Peterchev†

†These authors contributed equally to this work and share last authorship

*** Correspondence:** Lari M. Koponen: L.Koponen@bham.ac.uk

1 Supplementary Data

This supplement contains three supplementary figures and four supplementary tables.

Supplementary Figure 1 shows the flow diagram of the real-time TMS–EMG toolbox developed for the experiment, and **Supplementary Figure** 2 shows the user interface of the toolbox.

Supplementary Figure 3 shows all 48 input–output curves (8 participants with 3 muscle pairs each).

The four supplementary tables show, respectively, the coefficients of the four linear mixed-effects (LME) model fits and the corresponding contrasts between the muscles: **Supplementary Table 1** contains the model for the midpoints, **Supplementary Table 2** contains the model for the non-normalized slopes, **Supplementary Table 3** contains the model for the normalized slopes, and **Supplementary Table 4** contains the model for the inverse of the normalized recruitment spread.

2 Supplementary Figures and Tables

2.1 Supplementary Figures

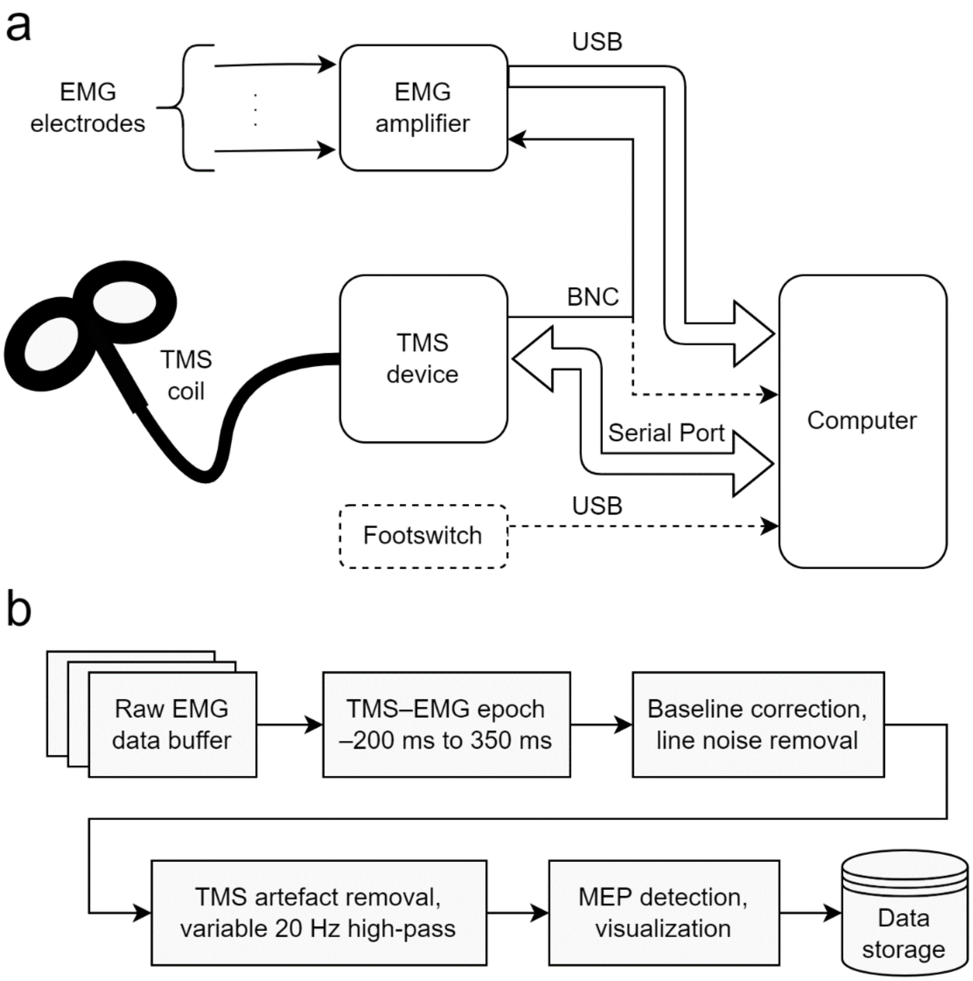

Supplementary Figure 1. Flow diagram of the TMS–EMG remote control toolbox developed for the experiment. The toolbox allows real-time analysis of multi-channel EMG acquired with BrainAmp ExG amplifier and automatic IO curve acquisition with randomized pulse order with a MagVenture MagPro TMS device. The toolbox contains a TMS device controller module that further logs the realized amplitude of each TMS pulse during the session; this controller can be used independently of the rest of the toolbox. The toolbox is available at https://github.com/L2K2/TMSEMG_toolbox under GPLv3 license. **(A)** Module diagram for the experiment, where an optional second USB keyboard (here, a USB footswitch) is used to start or pause IO curve acquisition. **(B)** Real-time signal-processing pipeline with approximately 400 ms pulse-to-screen latency.

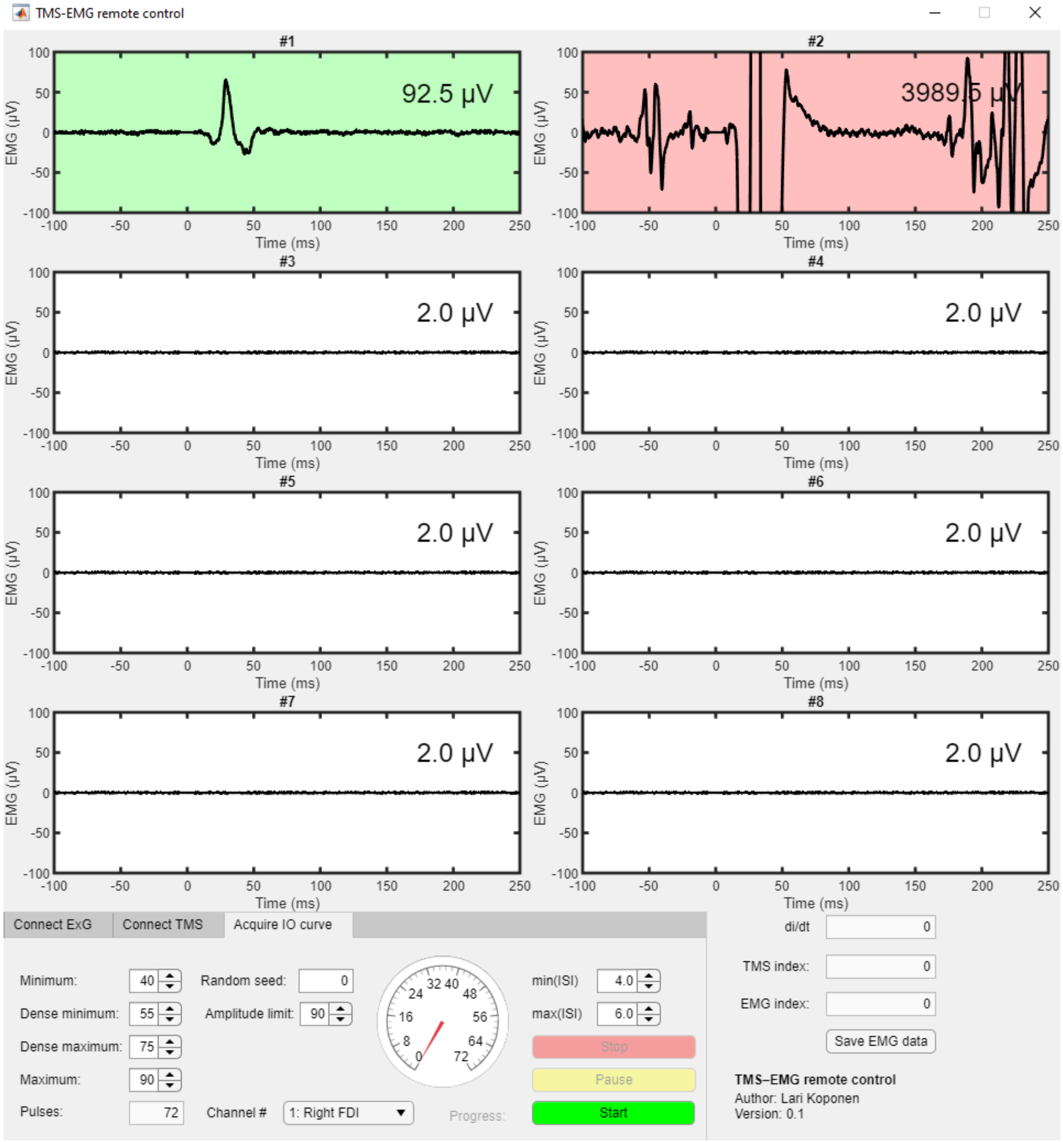

Supplementary Figure 2. User interface of the TMS–EMG remote control toolbox developed for the experiment. The top eight panels show the most recent TMS-evoked MEP response measured by the eight bipolar pairs of EMG electrodes. The shown data has been filtered as described in the work, including TMS-pulse artefact removal. In this composite image with representative example data, channel 1 shows an MEP that has been correctly identified (indicated by the green background) and channel 2 shows an MEP that has been correctly rejected due to excessive pre-activation (> 50 µV in the 100 ms time-window prior to TMS, indicated by the red background). Here, the other six channels are unconnected and show just noise. The controls near the bottom left allow connecting to the EMG amplifier, connecting to a TMS device, and the sampling of an IO curve, respectively. The monitor in the bottom right indicates the rate of change of coil current for latest pulse (here 0) and the number of trigger events received from the TMS and EMG devices (here 0), respectively. The two trigger counts will ideally increase at the same rate, however, in practice, either of the two devices can sometimes register an erroneous 'phantom' trigger event due to electromagnetic interference. To remove these errors, the output data contains timestamps that allow re-aligning the events (including alignment to neuronavigation data which is collected separately).

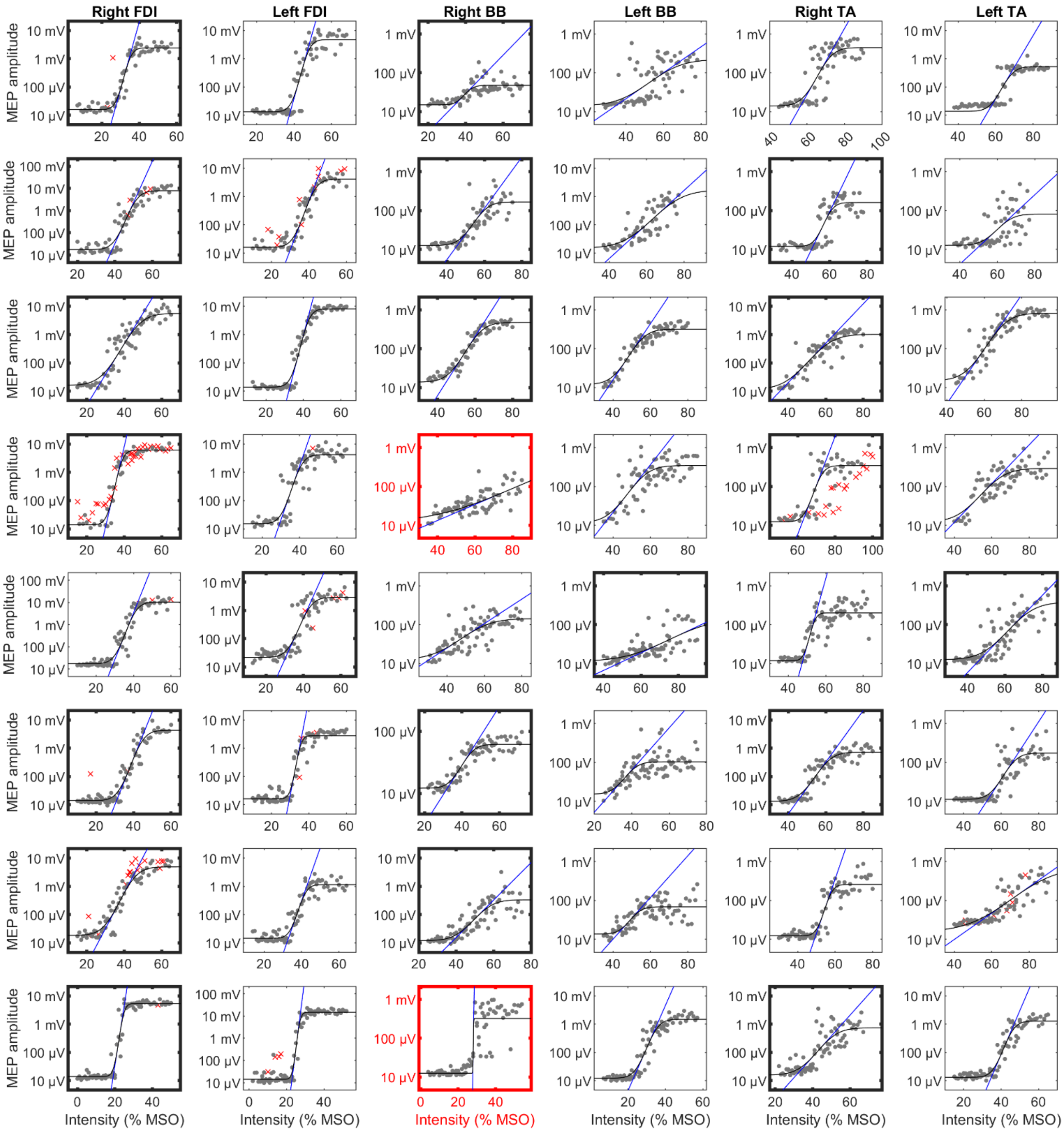

Supplementary Figure 3. All 48 IO curves. Each row depicts a participant, and each column a muscle. A bold box around a plot denotes the dominant side of the participant (handedness or footedness, respectively), and a red box a rejected IO curve. The IO curve of the right BB of participant 4 was rejected as the IO curve sampling does not span past predicted IO curve midpoint, and the IO curve of the right BB of participant 8 was rejected as the IO curve fit degenerated into a step function where the predicted upper plateau response lower less than the response of 9 out of 10 strongest pulses and 20 out of 25 strongest pulses.

2.2 Supplementary Tables

Supplementary Table 1A. LME model coefficients for midpoints. There were 42 observations, 4 fixed effects coefficients, 8 random effects coefficients, and 2 covariance parameters. The Akaike Information Criterion (AIC) was 299.08, the Bayesian Information Criterion (BIC) 309.51, the logarithmic likelihood –143.54, and the deviance 287.08. The random effects standard deviation estimate was 5.1273% MSO and the residual standard deviation 6.4204% MSO.

Parameter	β	SE	t	DF	p	95% CI
FDI	35.805	2.4213	14.788	38	$2.3962 \cdot 10^{-17}$	(30.904,40.707)
BB	48.443	2.5111	19.291	38	$3.1565 \cdot 10^{-21}$	(43.359,53.526)
TA	55.282	2.6526	20.841	38	$2.1452 \cdot 10^{-22}$	(49.912,60.652)
d – nd	1.5023	1.9955	0.75286	38	0.45617	(−2.5373,5.542)

Supplementary Table 1B. LME model contrasts for midpoints.

Parameter	p	F	$DF1$	$DF2$	Holm–Bonferroni	$p_{\text{Holm–Bonferroni}}$
FDI – BB	$4.5399 \cdot 10^{-6}$	28.539	1	38	2	$9.0798 \cdot 10^{-6}$
FDI – TA	$2.4831 \cdot 10^{-9}$	59.961	1	38	3	$7.4493 \cdot 10^{-9}$
BB – TA	0.012939	6.8028	1	38	1	0.012939
d – nd	0.45617	0.5668	1	38	1	0.45617

Supplementary Table 2A. LME model coefficients for non-normalized slopes. There were 42 observations, 4 fixed effects coefficients, 8 random effects coefficients, and 2 covariance parameters. The AIC was 170.87, the BIC 181.29, the logarithmic likelihood –79.433, and the deviance 158.87. The random effects standard deviation estimate was 0.92272 dB/% MSO and the residual standard deviation 1.438 dB/% MSO.

Parameter	β	SE	t	DF	p	95% CI
FDI	4.9211	0.48546	10.137	38	$2.3368 \cdot 10^{-12}$	(3.9383,5.9038)
BB	1.3026	0.50752	2.5667	38	0.014332	(0.27521,2.3301)
TA	1.6637	0.54074	3.0767	38	0.0038698	(0.56902,2.7583)
d – nd	−0.56916	0.44675	−1.274	38	0.2104	(−1.4735,0.33523)

Supplementary Table 2B. LME model contrasts for non-normalized slopes.

Parameter	p	F	$DF1$	$DF2$	Holm–Bonferroni	$p_{\text{Holm–Bonferroni}}$
FDI – BB	$4.1374 \cdot 10^{-8}$	46.694	1	38	3	$1.2412 \cdot 10^{-7}$
FDI – TA	$1.0646 \cdot 10^{-6}$	33.661	1	38	2	$2.1293 \cdot 10^{-6}$
BB – TA	0.54048	0.38151	1	38	1	0.54048
d – nd	0.2104	1.6231	1	38	1	0.2104

Supplementary Table 3A. LME model coefficients for normalized slopes. There were 42 observations, 4 fixed effects coefficients, 8 random effects coefficients, and 2 covariance parameters. The AIC was 446.68, the BIC 457.11, the logarithmic likelihood –217.34, and the deviance 434.68. The random effects standard deviation estimate was 0 dB and the residual standard deviation 42.769 dB.

Parameter	β	SE	t	DF	p	95% CI
FDI	164.97	10.692	15.429	38	$5.9847 \cdot 10^{-18}$	(143.32,186.61)
BB	54.371	11.47	4.7405	38	$2.9764 \cdot 10^{-5}$	(31.152,77.59)
TA	97.251	12.346	7.8769	38	$1.6545 \cdot 10^{-9}$	(72.257,122.24)
d – nd	−19.777	13.244	−1.4933	38	0.14362	(−46.588,7.0342)

Supplementary Table 3B. LME model contrasts for normalized slopes.

Parameter	p	F	$DF1$	$DF2$	Holm–Bonferroni	$p_{\text{Holm–Bonferroni}}$
FDI – BB	$2.0849 \cdot 10^{-8}$	49.746	1	38	3	$6.2548 \cdot 10^{-8}$
FDI – TA	0.00018268	17.189	1	38	2	0.00036535
BB – TA	0.015128	6.4745	1	38	1	0.015128
d – nd	0.14362	2.2299	1	38	1	0.14362

Supplementary Table 4A. LME model coefficients for inverse of normalized recruitment spread. There were 42 observations, 4 fixed effects coefficients, 8 random effects coefficients, and 2 covariance parameters. The AIC was 220.77, the BIC 231.2, the logarithmic likelihood –104.39, and the deviance 208.77. The random effects standard deviation estimate was 0 and the residual standard deviation 2.9051.

Parameter	β	SE	t	DF	p	95% CI
FDI	8.2777	0.72627	11.398	38	$7.97 \cdot 10^{-14}$	(6.8074,9.7479)
BB	5.9868	0.77906	7.6846	38	$2.9723 \cdot 10^{-9}$	(4.4096,7.5639)
TA	8.688	0.83862	10.36	38	$1.2661 \cdot 10^{-12}$	(6.9903,10.386)
d – nd	−0.72419	0.89959	−0.80503	38	0.42581	(−2.5453,1.0969)

Supplementary Table 4B. LME model contrasts for inverse of normalized recruitment spread.

Parameter	p	F	$DF1$	$DF2$	Holm–Bonferroni	$p_{\text{Holm–Bonferroni}}$
FDI – BB	0.037905	4.6264	1	38	2	0.075811
FDI – TA	0.71351	0.13683	1	38	1	0.71351
BB – TA	0.023518	5.5692	1	38	3	0.070553
d – nd	0.42581	0.64807	1	38	1	0.42581
